# Does adding an interbody cage in L4–L5 posterolateral fusion for degenerative spondylolisthesis and stenosis improve clinical outcome?

**DOI:** 10.1016/j.bas.2026.105926

**Published:** 2026-01-05

**Authors:** Enrico Aimar, Lucrezia Di Stefano, Federico Longhitano, Alberto Bona, Marco Meloni, Tommaso Alfiero, Federica Valente, Roberta Bonomo, Giulio Bonomo, Flavio Tancioni, Guglielmo Iess

**Affiliations:** a Department of Neurosurgery, Istituto Clinico Città Studi, Milan, Italy; b Columbus Clinic Center, Milan, Italy; c Department of Neurosurgery, Ospedale San Raffaele, Milan, Italy; d School of Medicine and Surgery, “Kore” University of Enna, Enna, Italy; e Department of Neurosurgery, Università Degli Studi di Catania, Catania, Italy; f Department of Neurosurgery, Thomas Jefferson University, Philadelphia, USA

**Keywords:** Degenerative spondylolisthesis, Lumbar fusion, Interbody cages, Elderly patients, Postoperative complications, Posterolateral fusion

## Abstract

**Introduction:**

As the population ages, L4–L5 degenerative spondylolisthesis is increasingly common. Posterolateral fusion was long standard; interbody cages are widely used for perceived higher fusion rates.

**Research question:**

Does adding an interbody cage to posterolateral fusion improve outcomes or reduce complications in elderly patients with grade I spondylolisthesis and severe stenosis?

**Materials and methods:**

We retrospectively studied 319 adults aged 60–85 who underwent single-level L4–L5 fusion (2011–2018) after failed conservative care. Patients received posterior lumbar fusion (PLIF, n = 155) or posterolateral lumbar fusion (PLF, n = 164). Primary outcomes were Oswestry Disability Index (ODI) change and complications over a median five-year follow-up; secondary outcomes were operative time, hospital stay, and transfusions. Multivariable analyses adjusted for age, sex, BMI, year of surgery, and sagittal alignment.

**Results:**

Functional improvement was similar (median ODI reduction ≈22 points; p = 0.97), and implant-related revision and revision-free survival did not differ. Cage use increased overall complications (24.5 % vs 7.9 %), prolonged surgery (125 vs 95 min) and hospital stay (4 vs 3 days), and raised transfusions (9.7 % vs 1.8 %), dural tears (11.6 % vs 2.4 %), and radicular deficits (6.5 % vs 1.2 %). The association between cage use and complications persisted after adjustment.

**Conclusion:**

In this elderly, low-grade L4–L5 degenerative spondylolisthesis cohort, adding a posterior interbody cage to instrumented posterolateral fusion did not improve 5-year disability but was associated with higher peri-operative morbidity and greater resource use.

## Highlights

• Adding an interbody cage did not improve 5-year disability after L4–L5 fusion.
• Cage use increased complications, blood transfusions, and hospital stay.
• Operations with a cage were longer and had more dural tears and nerve deficits.
• Reoperation rates were similar with and without a cage over five years.
• Fusion without an interbody cage may be a safer, simpler option for older patients.

## 1 Introduction

Spondylolisthesis—derived from the Greek spondyl (vertebra) and listhesis (slipping)—denotes anterior translation of one vertebra over another while the posterior neural arch and pars interarticularis remain intact (Pooswamy et al., 2017; Koslosky and Gendelberg, 2020). The slip can generate varying degrees of mechanical instability and narrow the central canal (Steiger et al., 2014; Omidi-Kashani et al., 2018).

Degenerative lumbar spondylolisthesis (DLS) is the most prevalent degenerative spinal disorder in older adults and is uncommon before age 50. Its overall incidence is ~ 4 % in the general population, with a female-to-male ratio near 6:1 (Steiger et al., 2014; Bydon et al., 2019). Although its pathogenesis is still a matter of debate among researchers, progressive disc degeneration is believed to increase facet-joint loading, producing hypertrophy that narrows the disc space and promotes “micro-instability.” Concomitant ligamentum-flavum hypertrophy and forward vertebral translation further compress the dural sac (Chen et al., 2011). A sagittally oriented facet configuration—common at L4–L5—amplifies risk at this level (Steiger et al., 2014; Chen et al., 2011).

Clinically, DLS manifests as mechanical low-back pain, neurogenic claudication from central stenosis, or radiculopathy from lateral-recess or foraminal compromise (Koslosky and Gendelberg, 2020; Bydon et al., 2019). Slip severity is most often graded with the Meyerding system, which guides surveillance and treatment (Koslosky and Gendelberg, 2020). Modern surgical goals therefore include reliable neural decompression plus restoration or maintenance of lumbar lordosis, disc height, and solid fusion. Yet heterogeneous patient presentations hamper universal guidelines and make procedure choice controversial (Tartara et al., 2023). Current treatment options span decompression alone or with fusion—posterolateral fusion (PLF) using pedicle-screw fixation, and numerous interbody approaches (PLIF, TLIF, ALIF, XLIF, facet-wedge fusion, minimally invasive variants) used singly or in combination (Steiger et al., 2014).

Although instrumented posterolateral fusion has long been the workhorse for low-grade DLS owing to its ability to stabilize the vertebrae while also providing direct spinal canal decompression, the use of interbody cages to achieve a circumferential (360°) fusion has risen steadily over the past two decades (Morse et al., 2022), likely because many surgeons believe it achieves better fusion rates, can increase foraminal height (McDonald et al., 2024), and restores segmental lordosis. In support of this rationale, randomized and meta-analytic data have shown that interbody fusion achieves higher radiographic fusion rates than PLF (Said et al., 2022; Challier et al., 2017; Levin et al., 2018; Dantas et al., 2022). However, these same studies have not consistently demonstrated superior restoration of segmental or global lordosis or better short-term clinical outcomes with cage use—for example, a Level-I randomized trial found no advantage in segmental lordosis with TLIF over PLF, and a recent meta-analysis reported only small changes in sacral slope without improvement in overall lordosis or symptoms (Challier et al., 2017; Dantas et al., 2022). In addition, the clinical value of achieving greater segmental lordosis remains uncertain; in a prospective TLIF cohort, failure to increase lumbar lordosis did not impair long-term outcomes (Bošnjak and Vengust, 2025). On the other hand, the utility of a cage may be offset by longer operative times, greater blood loss, and higher rates of peri-operative and long-term complications. Although overall complication rates appear comparable between techniques (Said et al., 2022), pooled evidence suggests a higher risk of neural injury with interbody approaches, likely related to root retraction during disc-space preparation (Dantas et al., 2022). Another interbody-specific risk is cage subsidence, which is associated with low vertebral bone density and posterior cage placement, and is more frequent in older patients (Amorim-Barbosa et al., 2022; Xie et al., 2022).

This trade-off is especially pertinent in the elderly, where diminished bone-healing capacity must be weighed against the morbidity of more complex, protracted procedures (Meinberg et al., 2019). Against this backdrop, we compared clinical outcomes in Grade I L4–L5 DLS treated with instrumented PLF with versus without an interbody cage, aiming to clarify the optimal strategy for this common scenario.

## 2 Materials and methods

### 2.1 Study design and patient selection

This retrospective observational study received institutional approval before data collection. From July 2011 to December 2018, a total of 349 adult patients underwent a single-level posterolateral arthrodesis (PLA) at L4–L5 for Grade I degenerative spondylolisthesis associated with symptomatic stenosis. Given our focus on a non-deformity population, patients with global sagittal malalignment were excluded a priori; the analysis therefore pertains to single-level L4–L5 DLS without pathological sagittal imbalance. All procedures were carried out at a single high-volume orthopedic center by the same primary surgeon. Because of a deliberate shift in surgical strategy, all operations performed between July 2011 and December 2014 incorporated both posterolateral fusion and an L4–L5 interbody cage, whereas procedures from January 2015 onward used instrumented posterolateral fusion alone. More in detail, eligibility criteria for inclusion were: (1) Meyerding Grade I degenerative spondylolisthesis at L4–L5 documented on plain radiographs; (2) lumbar scoliosis <5° on standing coronal radiographs; (3) absence of global sagittal malalignment (e.g., no pathological sagittal imbalance on imaging); (4) L4–L5 spinal canal stenosis of grade D according to the Schizas classification (Schizas et al., 2010); and (5) radiographic evidence of instability at L4–L5 on flexion-extension dynamic radiographs (defined by at least 4 mm of translational difference). Patients with prior lumbar surgery, presence of spondylolysis or pars defects, or other spinal levels requiring arthrodesis were excluded.

### 2.2 Operative techniques

Patients were treated with one of two lumbar fusion techniques:

1) Posterior lumbar interbody fusion (PLIF): From July 2011 to December 2014, 172 patients received a posterolateral fusion augmented by pedicle screws and rods, combined with an L4–L5 interbody cage. Bilateral transpedicular screws were inserted under intraoperative CT-based navigation (O-arm, Medtronic, USA), followed by partial or total laminectomy at L4–L5. An interbody cage was placed to restore disc height and promote fusion, and local bone graft was packed posterolaterally. The specific interbody device was a Medtronic CAPSTONE spacer (parallel profile, 0°), single cage at L4–L5 in all cases. Implants were selected by trialing to fit endplates; typical sizes at L4–L5 were 22–26 mm in length and 10–12 mm in height (device width 10 mm). The device's central graft chamber was filled with morselized autograft (∼0.5–0.9 mL depending on implant size), while the remaining graft (∼15–30 mL) was placed posterolaterally
2) Posterolateral fusion (PLF): From January 2015 to December 2018, 177 patients underwent a similar open posterior approach with pedicle screw fixation and posterolateral bone grafting, but no cage was placed in the intervertebral space.

In both techniques, decompression consisted of a bilateral laminectomy extended to the pedicles, with complete ligamentum flavum resection and undercutting of the rostral lamina when required by the pattern of stenosis; the superior spinoligamentous complex was preserved whenever feasible. After decompression, a midline posterior approach with bilateral pedicle-screw fixation was used. Local autograft was harvested from the spinous process and laminae (with additional morselized bone from the medial facets after undercutting). For posterolateral arthrodesis, the pars and both the medial segments of the transverse processes were decorticated, and the graft was packed along both intertransverse gutters.

Surgical indication was established after each patient failed conservative management, including medications, physical therapy, and at least three epidural steroid injections. In all cases, an intraoperative CT-based navigation system was used to guide pedicle screw placement.

### 2.3 Clinical and radiological data collection

Demographic and clinical information—age, sex, body mass index (BMI), and lumbar lordosis type (based on Roussouly classification)—were documented (Roussouly et al., 2005). All patients completed the Oswestry Disability Index (ODI) questionnaire preoperatively. Four operative-related factors were systematically recorded: operative duration (minutes), hospital length of stay (days), use of blood transfusion (yes/no), intra- and perioperative complications (e.g., dural tears, nerve root deficits, infections, hematomas) within 1 month post-surgery. Patients were followed for 5 years after their index procedure. In cases where a secondary surgery was required prior to the 5-year mark, data were collected up to the time of reoperation. At final follow-up or immediately before any revision procedure, the ODI questionnaire was repeated. Patients who could not attend an in-person review were contacted to complete the ODI via telephone or mail. All patients had preoperative plain standing radiographs (anteroposterior, lateral, and dynamic flexion-extension views) and an MRI scan of the lumbar spine. Instability was confirmed if flexion-extension radiographs demonstrated translation >4 mm at L4–L5. On follow-up, patients were encouraged to obtain repeat radiographs and MRI scans. However, only the ODI was mandatory for inclusion; imaging follow-up was collected when available to assess implant integrity, signs of adjacent pathology, or any complications such as pedicle screw pull-out, cage migration, or adjacent fractures.

Because long-cassette lateral radiographs sufficient to calculate pelvic parameters (pelvic incidence, pelvic tilt) were not consistently available across the entire retrospective time frame, these measures were not uniformly recorded. As a morphological proxy for spinopelvic alignment in this non-deformity cohort, we used the Roussouly classification, which recognises four morphotypes of lumbar lordosis by integrating sacral slope, apex level, and arc proportions (Roussouly et al., 2005): type I corresponds to a long, shallow curve with a low sacral slope and an apex at L5; type II reflects a flat, short lordosis with similarly low sacral slope; type III represents a “physiological” curvature, with sacral slope around 35°–45° and an apex at L4–L5; and type IV denotes a deep, steep curve characterised by sacral slope exceeding 45° and an apex at L3–L4. Lordosis type was included as a covariate in all multivariable models. Two independent, blinded observers performed all measurements, achieving excellent intra- and inter-reader agreement (κ > 0.80).

### 2.4 Outcome measures

The primary clinical outcome was the ODI score at 5 years (or at the time of any reoperation). Secondary outcomes included the incidence of revision procedures and the aforementioned operative-related parameters (length of surgery, hospital stay, transfusion rate, early and late complications). Any patient lost to follow-up was excluded from the final statistical analysis.

### 2.5 Statistical analysis

Continuous variables (e.g., age, BMI, surgical time, hospital stay, pre- and postoperative ODI) were checked for normality using the Shapiro–Wilk test and summarized as medians and interquartile ranges if non-normally distributed. Categorical variables (e.g., sex, transfusion requirement, complication occurrence) were expressed as frequencies and percentages. Group comparisons used the Mann–Whitney U, Pearson's chi-squared and linear-to-linear association tests, as appropriate.

To evaluate the association of surgical techniques (PLIF vs. PLF) with the change in ODI from baseline to follow-up, generalized linear models (GLMs) were employed, adjusting for potential confounders such as age, sex, BMI, and lordosis type. To examine the association between surgical technique and the likelihood of occurrence of at least one eventful complication, we fitted a generalized linear mixed model with binomial distribution and logit link. The dependent variable was eventful (yes/no). Fixed effects included surgical group (0 = posterolateral fusion with cage, 1 = without cage), age, sex, BMI, and lumbar lordosis type. We also added an operative period factor, defined as seven sequential calendar periods spanning the study years (July 2011–December 2018), to approximate the surgeon's learning curve. This variable was treated as a random intercept to account for potential variation in outcomes over time. Odds ratios (ORs) and 95 % confidence intervals were computed for each fixed effect.

The need for revision surgeries in the two groups was analyzed with a multivariate Cox proportional-hazards regression. For each patient we defined an event indicator (revision surgery = 1, no revision = 0) and a time-to-revision in months. Patients without revision by the end of follow-up were censored at 60 months, while those requiring revision contributed their actual time to revision (in months) derived from the database. The model included surgical group (with cage vs without), age, sex, BMI and Roussouly lordosis subtype as covariates. The proportional-hazards assumption was evaluated by examining scaled Schoenfeld residuals, and hazard ratios (HRs) with 95 % confidence intervals were reported. All analyses were performed using SPSS (Version 27.0, IBM Corp., Armonk, NY, USA), with significance set at p < 0.05.

## 3 Results

A total of 349 patients who underwent L4–L5 lumbar fusion were reviewed, of whom 172 (49.3 %) received PLIF and 177 (50.7 %) PLF. 30 patients (8.6 %) were lost to follow-up: 17/172 PLIF patients (9.9 %) and 13/177 PLF patients (7.3 %). The final analysis therefore included 319 patients overall: 155 (48.6 %) in the PLIF group and 164 (51.4 %) in the PLF group. At baseline (Table 1), the two cohorts were clinically and radiographically comparable: the median age was 76 years (IQR 7) in PLIF vs. 77 years (IQR 5) in PLF (U = 11 667.0; *p* = 0.204; Mann–Whitney test), median BMI was 26 (IQR 3) vs. 27 (IQR 3; U = 13866.500; *p* = 0.157; Mann–Whitney test), and median baseline ODI was 30 (IQR 3) in both groups (U = 12 843.0; *p* = 0.871; Mann–Whitney test). Similarly, the distribution of lordosis subtypes did not differ significantly between PLIF and PLF (χ^2^ (1) = 0.41; *p* = 0.525; linear-by-linear association).

**Table 1:** Descriptive statistics of the two groups at baseline.

Parameter	PLIF group	PLF group	p-value
**Age, years** [median; IQR]	76; 7	77; 5	0.204^a^
**Sex**, n [%]	Females = 88 [56.8 %]	Females = 97 [59.1 %]	0.668^b^
	Males = 67 [43.2 %]	Males = 67 [40.9 %]	
**BMI** [median; IQR]	26; 3	27; 3	0.157^a^
**ODI at baseline** [median; IQR]	30; 3	30; 3	0.871^a^
**Lordosis category**, n [%]	Type 1 = 20 [12.9 %]	Type 1: 19 [11.6 %]	0.525^c^
	Type 2 = 49 [31.6 %]	Type 2: 59 [36.0 %]	
	Type 3 = 59 [38.1 %]	Type 3: 65 [39.6 %]	
	Type 4 = 27 [17.4 %]	Type 4: 21 [12.8 %]	

P values that are statistically significant are shown in bold.

BMI Body Mass Index, PLIF Posterior lumbar interbody fusion, PLF Posterolateral lumbar fusion, ODI Oswestry Disability Index, IQR, interquartile range.

a Mann-Whitney *U* test.

b Chi-squared test.

c Linear-by-linear association.

At a median 5‐year follow‐up, functional recovery was similar: the median ODI improved in both groups (Table 2), and the distributions of ODI change did not differ on univariate analysis (*U* = 12 596; *p* = 0.970; Mann–Whitney test). In a multiple linear regression model adjusting for BMI, age, sex, and lumbar‐lordosis subtype, surgical group remained a non‐significant predictor of ODI improvement (B = −0.10; 95 % CI ‒1.82 to 1.63; *t* = −0.11; *p* = 0.910) (see Table 3).

**Table 2:** Descriptive statistics of the two groups at 5 years follow-up.

Parameter	PLIF group	PLF group	p-value
**ODI at follow-up** [median; IQR]	7; 12	7; 13	0.832^a^
**ODI difference** [median; IQR]	22; 10	22.5; 10	0.998^a^
**Operative time** [median; IQR]	125; 10	95; 10	**< 0.001**^a^
**Hospital stay** [median; IQR]	4; 1	3; 1	**< 0.001**^a^

P values that are statistically significant are shown in bold.

PLIF Posterior lumbar interbody fusion, PLF Posterolateral lumbar fusion, IQR, interquartile range.

a Mann-Whitney *U* test.

**Table 3:** Complication frequencies of the two groups at 5 years follow-up.

Parameter	PLIF group	PLF group	p-value
**Eventful**, n [%]	38 [24.5 %]	13 [7.9 %]	**< 0.001**^a^
**Durotomy,** n [%]	18 [11.6 %]	4 [2.4 %]	**0.003**^a^
Repaired, n [%]	12 [7.7 %]	3 [1.8 %]	**0.026**^a^
Unrepaired, n [%]	6 [3.9 %]	1 [0.6 %]	0.110^a^
**Radicular deficit**, n [%]	10 [6.5 %]	2 [1.2 %]	**0.014**^a^
**Spondylodiscitis,** n [%]	5 [3.2 %]	2 [1.2 %]	0.222^¶^
**Wound dehiscence,** n [%]	7 [4.5 %]	1 [0.6 %]	**0.026**^a^
**Drained haematoma,** n [%]	5 [3.2 %]	5 [3.0 %]	0.928^a^
**Revision surgery,** n [%]	13 [8.4 %]	12 [7.3 %]	0.722^a^
Hardware failure, n [%]	6 [3.9 %]	4 [2.4 %]	0.463^a^
ASD, n [%]	7 [4.5 %]	8 [4.9 %]	0.879^a^
**Trasfusion,** n [%]	15 [9.7 %]	3 [1.8 %]	**0.002**^a^

P values that are statistically significant are shown in bold.

PLIF Posterior lumbar interbody fusion, PLF Posterolateral lumbar fusion, ASD, Adjacent Segment Disease.

a Chi-squared test.

**Fig. 1:**
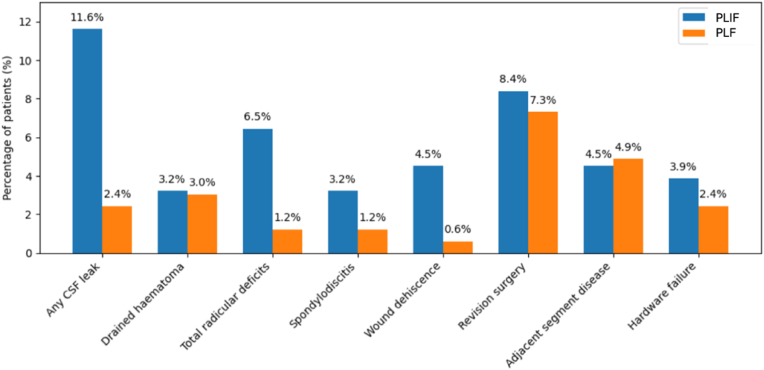
Bar chart graph illustrating the distributions of specific complication rates between groups. PLIF, posterior lumbar interbody fusion; PLF, posterolateral lumbar fusion.

Early morbidity, however, favored the non‐cage technique (see Fig. 1 and Table 3). Patients who underwent interbody fusion had longer operative times (125 vs. 95 min; U = 24 716.5; *p* < 0.001) and a significantly greater overall complication rate (24.5 % vs. 7.9 %; χ^2^ (1) = 15.12; *p* < 0.001; Chi-Square test). Transfusion requirements were also higher in the PLIF group (9.7 % vs. 1.8 %; χ^2^ (1) = 7.80; *p* = 0.002; Chi-Square test). Length of stay differed significantly between techniques: the cage group had a median of 4 days (IQR 1) versus 3 days (IQR 1) in the non-cage group (U = 18 752; p < 0.001; Mann–Whitney test).

Dural tears mirrored this pattern. In total, 11.6 % of PLIF patients experienced intraoperative durotomies versus 2.4 % in PLF (χ^2^ (1) = 9.06, p = 0.003; Chi-Square test). Of these, 7.7 % were successfully repaired (vs. 1.8 % in PLF; χ^2^ (1) = 4.97, p = 0.026; Chi-Square test), while 3.9 % remained unrepaired (vs. 0.6 % in PLF; χ^2^ (1) = 2.58, p = 0.11; Chi-Square test). Multivariate logistic regression confirmed that cage use independently increased the odds of durotomy repair (B = −1.41; OR = 0.25; *p* = 0.033), though the excess of unrepaired CSF leaks did not reach significance after covariate adjustment (B = −1.91; OR = 0.15; *p* = 0.082).

Radicular deficits were also more common in the cage group (6.5 % vs 1.2 %; χ^2^ (1) = 4.67, *p* = 0.031; Chi-Square test). There were no significant differences in postoperative spondylodiscitis (5 vs 2 cases; χ^2^ (1) = 0.71, *p* = 0.40; Chi-Square test), drained haematomas (5 cases in each group) or revision surgeries (13 vs 12 cases; χ^2^ (1) = 0.02, *p* = 0.88; Chi-Square test). Wound dehiscence occurred in 7 cage patients (4.5 %) and 1 non-cage patient (0.6 %), a trend that nevertheless did not reach significance (χ^2^ (1) = 3.50, *p* = 0.06; Chi-Square test).

Analysing complications altogether, we defined an “eventful” case as a patient experiencing at least one intra- or post-operative complication. A generalized linear mixed model adjusting for age, BMI, sex, lordosis type and including operative period as a random intercept showed that the surgical group remained a strong predictor of complications. Patients treated with a cage had approximately four-fold higher odds of experiencing a complication compared with those without a cage (adjusted OR = 3.8; 95 % CI 1.9 to 7.6; *p* < 0.001). None of the other covariates reached statistical significance (all p ≥ 0.12). The variance component for the operative-period random intercept was extremely small (<10^−6^), indicating that calendar period—and, by extension, the surgeon's learning curve—had a negligible effect on complication risk. These findings suggest that, even after accounting for patient demographics, lordosis subtype and potential improvements in surgical technique over time, the use of an interbody cage was independently associated with a higher likelihood of complications.

**Fig. 2:**
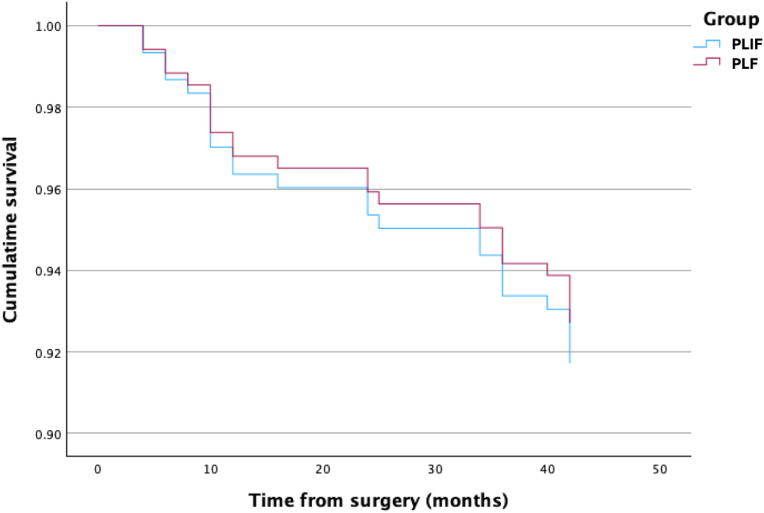
Kaplan–Meier curves of revision-free survival after L4–L5 fusion. Outcomes are virtually identical for posterolateral arthrodesis with a cage (PLIF, blue) and without a cage (PLF, red), indicating no meaningful difference in the need for re-operation over 5 years.

Re-operations were infrequent (25/319 patients, 7.8 % overall) and evenly distributed between groups: 13 cases in the PLIF arm (8.4 %) versus 12 cases in the PLF arm (7.3 %) (χ^2^ (1) = 0.13; *p* = 0.722; Chi-Square test). Hardware failure accounted for 6 cases in PLIF (3.9 %) and 4 cases in PLF (2.4 %) while ASD prompted 7 revisions in PLIF (4.5 %) and 8 revisions in PLF (4.9 %). None of these differences reached statistical significance. Implant-related revision was further assessed using the Cox model described above. After adjusting for age, sex, BMI and lordosis subtype, the hazard of revision did not differ between the cage and non-cage groups (HR = 0.88; 95 % CI 0.40–1.93; Wald χ^2^ = 0.11; p = 0.74; see Fig. 2).

## 4 Discussion

As life expectancy continues to rise, spinal surgeons are confronted with an expanding prevalence of degenerative disorders—including spinal stenosis, spondylolisthesis, disc herniation and age-related deformity (Atlas et al., 2005). DLS most commonly involves the highly mobile L4–L5 segment and is Grade I in roughly 85 % of cases, a severity that rarely demands aggressive reduction (Kalichman et al., 2008; Hey et al., 2024; Macki et al., 2015). Although segmental instability predominantly elicits axial back pain, disability is driven chiefly by neural compression in the central canal or foramina, manifesting clinically as neurogenic claudication or radiculopathy (Frymoyer, 1994). Accordingly, surgical treatment focuses on adequate neural decompression and attainment of a solid fusion, while the value of routinely restoring segmental and global sagittal alignment in single-level constructs remains controversial, with studies reaching conflicting conclusions about the impact of segmental lumbar lordosis on clinical outcome (Bošnjak and Vengust, 2025; Lian et al., 2013; Koucheki et al., 2023; Rhee et al., 2017; Aoki et al., 2022; Hsieh et al., 2018).

Growing evidence has shown that for DLS, fusion combined with decompression outperforms decompression alone even in the elderly, where single-level constructs do not appear to incur excess morbidity (Ghogawala et al., 2016; Herkowitz et al., 1991; Aimar et al., 2022). Debate instead centers on the optimal fusion strategy. Posterior interbody fusion techniques (PLIF, TLIF) and posterolateral fusion (PLF) remain the mainstays among myriad options that now include XLIF, ALIF, facet-wedge fusion and minimally invasive variants (Spiker et al., 2018; Aimar et al., 2021; Iess et al., 2024). This is because the posterior approaches permit optimal direct decompression together with fusion. Historically PLF dominated, but interbody constructs have gained growing popularity: American Board of Orthopaedic Surgery case logs show interbody use in DLS rising from 14 % (1999–2001) to 32 % (2009–2011), so that by 2011 PLF and PLF + interbody were performed in similar proportions (Morse et al., 2022). A recent AO Spine survey reached a similar conclusion—only one surgeon in ten favored instrumented PLF alone for a representative Grade-I slip, whereas nearly one-third chose an open TLIF.

Proponents of interbody techniques cite superior anterior-column support and consistently higher fusion rates (Campbell et al., 2020), yet head-to-head evidence seldom shows better functional outcomes and invariably documents longer procedures and greater peri-operative risk. Moreover, biotechnological advances have transformed pedicle screw–rod constructs over the past two decades, making today's systems markedly stronger, more fatigue-resistant and far less prone to breakage or loosening than the devices on which much of the earlier literature is based. Modern screws employ larger outer diameters, thicker cores, and optimized thread geometry, features that improve pull-out and fatigue performance; for example, increasing diameter from 6 mm to 8–10 mm increases fatigue load by roughly 35–50 %, particularly in low-density bone (Cho et al., 2010; Viezens et al., 2021). In addition, polyaxial heads—which replaced rigid monoaxial designs in the late 1990s—eliminate rod–screw alignment stress and have dramatically reduced clinical loosening (Wang et al., 2017). For osteoporotic bone, fenestrated screws allowing controlled cement augmentation are also now available and have been shown to reduce loosening and revision rates several-fold (Vasavada et al., 2025).

In this context we decided to conduct our article, to test the common conception that for elderly population with a single-level grade I DLS, interbody fusion is required. Importantly, while most of the literature on performance metrics between PLF and interbody fusion often includes different types of spondylolisthesis (degenerative, isthmic and traumatic), our study included only degenerative spondylolisthesis, and this was reflected by the high median age of the cohort (76 years). While we did not evaluate long-term fusion rates in our paper, in our homogeneous cohort, adding an interbody cage failed to deliver any functional advantage: five-year ODI improvement was very similar, and surgical technique was not an independent predictor of outcome in multivariable modelling. Because ODI was collected systematically only pre-operatively and at 5-year follow-up, we cannot exclude transient differences in early postoperative recovery between techniques. Moreover, ODI—although widely used and validated as a measure of disability in lumbar fusion—may not capture all dimensions of recovery. In our cohort, however, both groups improved by approximately 22 ODI points, and the adjusted between-group difference in ODI change was close to zero (B = −0.10; 95 % CI ‒1.82 to 1.63), making a clinically meaningful long-term disparity in disability between PLIF and PLF unlikely, even if subtler differences at earlier time intervals may have gone undetected.

By contrast, cage use predictably lengthened the operation (≈30 min), prolonged hospital stay by a full day, but, more importantly, trebled the overall complication rate, and increased transfusion requirements five-fold. Durotomy, radicular deficit, and wound-healing problems were all significantly more common in the cage group, yet the rates of reoperations due to either hardware failure and adjacent segment disease were comparable in the two cohorts, undermining any argument that the extra morbidity is offset by superior durability. In other words, within this elderly, low-grade population, posterolateral fusion alone provided comparable medium-term clinical recovery while being associated, in our cohort, with shorter operations, shorter hospital stays, and a lower observed peri-operative complication burden and transfusion rate. Beyond fusion rates, we acknowledge that pelvic parameters and segmental L4–L5 lordosis were not uniformly collected across the retrospective period. In single-level L4–L5 DLS, interbody techniques can modestly improve alignment surrogates (e.g., sacral slope/foraminal height), yet randomized trials and meta-analyses show no consistent advantage in ODI or pain over PLF (McDonald et al., 2024; Challier et al., 2017; Levin et al., 2018; Dantas et al., 2022; Caelers et al., 2024). As previously stated, although some series suggest that insufficient segmental lordosis may unfavorably affect mechanics and adjacent-segment risk after PLIF/TLIF (Chen et al., 2011; Lee et al., 2014), other cohorts (including long-term TLIF) report good outcomes despite minimal lordosis gain (Bošnjak and Vengust, 2025), so the clinical impact of routinely restoring greater segmental lordosis in single-level constructs remains uncertain. Within our non-deformity cohort after excluding global sagittal malalignment and adjusting for Roussouly morphotype, adding an interbody device did not improve functional recovery versus instrumented PLF alone.

Likewise, a 2022 systematic review of eight studies (616 patients) reported no meaningful differences in Oswestry Disability Index (ODI), pain scores, clinical satisfaction, complications, re-operations and blood loss—calling into question any tangible clinical benefit of routine cage placement (Said et al., 2022). Randomized trials reinforce this pattern. Farrokhi et al. (88 patients, 24-month follow-up) observed marginally better ODI and VAS improvements after PLF than after PLIF, despite lower radiographic fusion rates (Farrokhi et al., 2018). Cheng et al. (144 patients, mixed levels and slip grades) found similar ODI scores but significantly more hardware failure and non-union in the PLF cohort; the study's heterogeneity, however, tempers interpretation (Cheng et al., 2009). Høy et al. (100 patients, 2-year follow-up) detected no advantage for TLIF over PLF on multiple patient-reported measures, and early-complication profiles were equivalent (Høy et al., 2013).

In contrast, Macki et al. (2015) reported different findings in a large, single-centre retrospective study of 103 single-level fusions with a mean follow-up of 66 months (Macki et al., 2015). Although interbody constructs achieved greater slip correction (mean Taillard reduction 13.1 % vs 5.7 %; p < 0.001), early symptom relief was comparable and even trended in favour of PLF. Nevertheless, re-operation for degenerative progression occurred in 29 % of PLF patients but only 9 % of interbody patients (p = 0.011); multivariable analysis identified non-interbody fusion as the dominant predictor of re-operation (adjusted OR 0.18 for PLF + PLIF/TLIF vs PLF, conferring an 82 % risk reduction; p = 0.007). All cases of pseudarthrosis or hardware failure also clustered in the PLF group (9 % vs 0 %; p = 0.043). Although these authors suggest that interbody cages may enhance fusion durability, other evidence indicates that the added rigidity of an interbody construct, compared with fusion limited to the posterior elements, increases mechanical stress on neighboring segments and, over long-term follow-up, may increase adjacent-segment stresses.

Several observational series have reported higher rates of radiographic and clinical adjacent-segment degeneration and re-operation following PLIF or TLIF compared with posterolateral fusion alone, although results vary and may reflect differences in patient selection and fusion levels (Lee et al., 2014; Rahm and Hall, 1996).

Cost-effectiveness analyses also suggest that a cage adds cost more readily than value in grade-I DLS. In a 137-patient single-centre series modelled over 3.5 years, Bydon et al. found that PLIF/TLIF raised mean direct costs by roughly US $6000 per case yet delivered no statistically significant Quality-Adjusted Life Year (QALY) gain; the resulting incremental cost-effectiveness ratio (ICER) was ≈ US $71,000/QALY—above the traditional US $50 k willingness-to-pay threshold and therefore economically unattractive (Bydon et al., 2015). A larger five-year billing audit by Lyons et al. (233 cases) likewise showed steeper hospital charges for interbody constructs (US $51.1 k) than for either uninstrumented (US $36.9 k) or instrumented PLF (US $45.7 k), without any improvement in hospital margins, highlighting a net negative effect on resource utilisation (Lyons et al., 2019). More recent modelling tempered those findings but only under favourable assumptions: a 60-month Markov analysis restricted to Medicare fees calculated an average cost of US $29,511 per QALY gained for PLIF/TLIF, comfortably below the US $100 k societal threshold, yet still contingent on lower implant prices and the projection that reduced late re-operations offset higher index costs (Yee et al., 2024).

Taken together, our results, when viewed alongside the broader literature, raise questions about the current tendency to offer interbody fusion as the default procedure for elderly patients with low-grade DLS. In this population, a posterolateral fusion alone appears to provide comparable medium-term functional improvement, with shorter operative times, fewer peri-operative risks, and lower costs. By contrast, interbody fusion should be considered the gold standard for younger individuals, for higher-grade or translationally unstable slips, or whenever achieving maximal fusion integrity is essential.

The present study's strengths include its sizeable single-surgeon cohort, uniform indications, and standardised peri-operative care, all of which limit selection bias. Important limitations remain. First, the retrospective design and voluntary imaging follow-up may introduce unmeasured confounding. Second, temporal clustering of techniques (PLIF in 2011–2014, PLF in 2015–2018) raises the possibility of a learning-curve effect. We addressed this analytically by including operative period as a random intercept, which showed negligible variance (<10^−6^), arguing against a major calendar-period effect on complications; nonetheless, residual, unmeasured aspects of experience cannot be fully excluded. Third, our interbody cohort underwent a posterior lumbar interbody fusion (PLIF) approach rather than TLIF. Comparative evidence suggests that, relative to PLIF, TLIF may modestly favour peri-operative risk—showing shorter operative time, lower blood loss, and fewer dural tears—while achieving similar fusion rates and patient-reported outcomes (Zhang et al., 2014; de Kunder et al., 2017); conversely, a recent multicentre randomized non-inferiority trial in single-level spondylolisthesis found no differences between TLIF and PLIF in disability improvement or complication rates, and a contemporary bicentric cohort using a standardised complication classification likewise reported broadly similar overall complication profiles between techniques [Caelers et al.; Mehren et al.] (Caelers et al., 2024; Mehren et al., 2025). Nevertheless, we still believe that our observation of higher morbidity with interbody fusion should be interpreted as PLIF-specific and may not generalise to TLIF (including minimally invasive TLIF).

## 5 Conclusion

In elderly patients with grade I L4–L5 degenerative spondylolisthesis and severe stenosis who are selected for single-level fusion, instrumented posterolateral fusion without an interbody cage achieved similar 5-year ODI improvement to posterolateral fusion augmented by a cage, but in our cohort was associated with shorter operative times and fewer complications. Within the constraints of this retrospective single-centre study—and in the absence of systematic data on radiographic fusion, segmental lordosis and other alignment parameters—these findings suggest that routine cage insertion may not be necessary for this specific elderly, low-grade population; whether interbody devices confer advantages in younger patients, higher-grade or more unstable slips, multilevel fusions or minimally invasive approaches remains an important question for future prospective studies.

## Ethical considerations

All patients provided written informed consent before surgical treatment. Data were collected and analyzed confidentially in accordance with national legislation and institutional guidelines. No additional risks or interventions were introduced as part of this retrospective study.

## Consent for publication

Not applicable. No individual, identifiable patient data (images, videos, quotations) are presented.

## Author contributions (CRediT taxonomy)

Enrico Aimar (EA): Conceptualization; Methodology; Investigation. Lucrezia Di Stefano (LDS): Investigation; Data curation; Writing – original draft. Federico Longhitano (FL): Visualization; Writing – review & editing. Alberto Bona (AB): Investigation; Resources; Writing – review & editing. Marco Meloni (MM): Investigation; Project administration; Writing – review & editing. Tommaso Alfiero (TA): Investigation; Data curation; Writing – review & editing. Federica Valente (FV): Supervision; Writing – review & editing. Roberta Bonomo (RB): Validation; Data curation; Writing – review & editing. Giulio Bonomo (GB): Methodology; Supervision; Writing – review & editing. Flavio Tancioni (FV): Supervision; Writing – review & editing; Guglielmo Iess (GI): Conceptualization; Methodology; Formal analysis; Supervision; Writing – original draft.

## Declaration of generative AI and AI-assisted technologies in the writing process

During the preparation of this work, the authors used OpenAI's ChatGPT to improve grammar and readability and, where useful, to adjust wording and formatting to align the manuscript with the journal's author guidelines. After using this tool, the authors reviewed and edited the content as needed and take full responsibility for the content of this publication.

## Funding

This research received no specific grant from funding agencies in the public, commercial, or not-for-profit sectors.

## Declaration of competing interest

The authors declare that they have no known competing financial interests or personal relationships that could have appeared to influence the work reported in this paper.

## Data Availability

De-identified data that support the findings of this study are available from the corresponding author upon reasonable request and subject to institutional approvals and data-sharing agreements. Requests should be directed to guglielmoiess@gmail.com.
